# *Lactobacillus salivarius*, a Potential Probiotic to Improve the Health of LPS-Challenged Piglet Intestine by Alleviating Inflammation as Well as Oxidative Stress in a Dose-Dependent Manner During Weaning Transition

**DOI:** 10.3389/fvets.2020.547425

**Published:** 2020-12-16

**Authors:** Zeyang Sun, Haihua Li, Yupeng Li, Jiayun Qiao

**Affiliations:** ^1^Tianjin Key Laboratory of Conservation and Utilization of Animal Diversity, College of Life Sciences, Tianjin Normal University, Tianjin, China; ^2^Tianjin Key Laboratory of Agricultural Animal Breeding and Healthy Husbandry, College of Animal Science and Veterinary Medicine, Tianjin Agricultural University, Tianjin, China; ^3^College of Life Sciences, Tianjin Institute of Animal Husbandry and Veterinary Medicine, Tianjin, China

**Keywords:** *L. salivarius*, inflammatory response, oxidative stress, post-weaned piglets, LPS

## Abstract

Intestinal health is a critical issue for piglets during their weaning transition period. Previous reports have emphasized the promise of distinct probiotics in improving the enteric health. Here in this research, a newly isolated *Lactobacillus salivarius* strain was pretreated to Lipopolysaccharide (LPS)-challenged piglets and its association with integrity of the intestinal barrier coupled with effective dosage were expected to be signified. In the present study, 72 piglets (Landrace × Yorkshiere × Duroc) were randomly allotted to four groups, each group with six replicates. The subjects in the control group were provided with basal diet while those in other tested groups with extra 0.05, 0.1, and 0.2% *L. salivarius*, respectively. Fourteen days later, LPS was intraperitoneally injected and sodium pentobarbital was then delivered to euthanize those LPS-challenged piglets. An increase of average daily gain and body weight along with an apparent decline of diarrhea rate were observed in *L. salivarius*-treated groups. Both 0.1 and 0.2% *L. salivarius* supplement in total diet had the capability to markedly elevate levels of CAT, GSH-Px, SOD, anti-inflammatory cytokine from the serum as well as tight junction proteins (Claudin-1, Occludin, and ZO-1) extracted from intestine in LPS-challenged piglets. These changes were accompanied by the obvious downregulation of D-lactic acid, DAO, MDA and pro-inflammatory mediators in the serum, including IL-1β, IL-6, IFN-γ, and TNF-α. Meanwhile, the expression levels of TLR2 and TLR4 in spleen and mesenteric lymph nodes were significantly lower whereas the oxidation-related gene, *ho-1* was up-regulated with *L. salivarius* administration. Our findings suggested that relatively high dose *L. salivarius* (0.1–0.2%) could regulate the progression of inflammatory response and oxidative stress when individuals were exposed to LPS, thus probably offering valuable assistance in restoring barrier function and improving overall performance.

## Introduction

Weaning, coming after lactation, is a critical and stressful stage in which piglets have to undergo various challenges and makes those post-weaned piglets more susceptible to some intestinal diseases. For instance, bacterial diarrhea is quite frequent among piglets especially 2 weeks after weaning, which is commonly known as post-weaning diarrhea (PWD). Previous studies have stated that PWD is closely correlated with *Escherichia coli* (*E. coli*) infection from the environment (1) and those affected individuals tend to develop yellow watery diarrhea and suffer from dehydration (2). Meanwhile, an abrupt separation from lactating sows is another stress factor for those young piglets to handle and corticotropin-releasing factor (CRF) was previously suggested to stimulate stress-induced intestinal dysfunctions (3, 4). Since the resulting elevated rate of mortality, growth retardation and requirement of extra medication have adverse influence on the swine industries; broader attentions with respect to porcine intestinal health have been constantly paid.

An epithelial barrier is maintained between the luminal enclosure of the intestine and the internal environment of the body (5). The role of this barrier is not only restrained in preventing harmful entities, such as dietary antigens and pathogens, from the gut lumen into other tissue compartments or circulation (6), but also has the capability to permit the nutrient absorption and waste exclusion (7). The nutrient absorption is initiated either through transmembrane pathway, generally the solute transport, or paracellular one (8). Functional analysis indicates that the selective paracellular permeability could be regulated by tight junctions, the multi-protein complexes located in the lateral space between individual cells (9). Tight junctions consist of a branching network of proteins, among which Occludin (10) and Claudin (11) are trans-membrane proteins and scaffolding ZO-1 anchors the cytoskeleton to the trans-membrane domains of Occludin (12). All these integral proteins are suggested to be dynamic (13) and have pivotal roles in regulating barrier junction and even cell survival (10–12).

A bunch of physiological and pathological factors could contribute to the dysfunction of intestinal barrier. For example, the apoptosis of epithelial cells is associated with tight junction disruptions since both Occludin and ZO-1 might go through cleavage with distinctive manners (14, 15). Pro-inflammatory cytokines mediated by infections or traumas appear with the dissembling of tight junction organization and structure, and thus lead to an increased permeability (16, 17). This epithelial breach is responsible for the discontinuous intestinal barrier and makes functional substances in gut penetrate into the portal blood, such as diamine oxidase (DAO), a digestive enzyme, and D-lactic acid (18). Under this terrible circumstance, bacteria [mainly Firmicutes and Bacteroidetes (19)] and food antigens are more likely to be translocated from the lumen into the mesenteric lymph nodes (MLNs), blood stream and other organs like the spleen and liver. Obviously, this bacterial passage is a contributor to the immune response and inflammatory cytokines will be simultaneously released. In this case, perpetuation of chronic inflammation in intestinal site is likely and probably keeps giving rise to a vicious cycle.

Besides, some studies have reported that post weaning is a potent activator of oxidative stress, a condition where the imbalance between free radicals, or also called reactive oxygen species (ROS) and the antioxidants occurs. These free radicals, partly generated as the byproducts of mitochondrial respiration (20), are normally regarded as the main cause of oxidation because of their capability to easily react with other molecules, which is either beneficial or harmful for bodies. As a matter of fact, ROS with non-toxic quantity plays a crucial role in maintaining the cellular functions and supporting physiological metabolism, such as cell differentiation, proliferation and viability (21, 22). However, overproduction of ROS could put risks to an assortment of cellular abnormalities, including but not limited to biomacromolecule damage, cell apoptosis, and oxidative stress as mentioned before. Therefore, antioxidants provided by cells offer an electron to free radicals which could then be inactivated and eradicated; thus, they usually act as the oxidant defensive system to antagonize the disturbed homeostasis caused by oxidative burst. These include the use of Superoxide dismutase (SOD), catalase (CAT), and Glutathione peroxidase (GSH-Px) to counteract the excessive amount of ROS, all of which could be considered as trustworthy parameters for oxidative stress. The oxidant degradation of lipids results in a process called lipid peroxidation during which the generated lipid peroxides could be decomposed into Malondialdehyde (MDA). As a biochemical product of lipid peroxidation, it represents another reliable indicator for fat oxidant status in cells, and could indirectly reveal the damage of cells (23).

In this study, we took advantage of Lipopolysaccharide (LPS), a major component of cell wall from the Gram-negative bacteria, to primarily establish a bacteria-induced piglet model. Toll-like receptors (TLRs) are believed to participate in the innate immune system of the host by recognizing a diversified array of foreign antigens known as pathogen associated molecular patterns (PAMPs) (24). This identification has been designated as a potent inducer of activating inflammatory cytokines and chemokines (25). Many groups have manifested that TLR2 and TLR4 are able to sense and bind LPS, generally acting as crucial mediators of distinct cellular responses under this pathogenic condition (26).

Despite the fact that probiotics, a common type of living microbes, have been routinely involved in piglet diet in an attempt to boost pig health and performance, the administration of probiotics and its mechanisms have still been an uncharted territory in practice. The proper application of probiotics has been reported to have connection with gut improvement and superior nutrient utilization (27). Plentiful experiments *in vivo* or *in vitro* have pronounced the protective strategies of different Lactobacillus species against the intestinal dysfunction of piglets. However, *L. salivarius* is a newly separated strain and the efficacy of this particular strain as well as the appropriate dosage still remain ambiguous when aiming to combat digestive issues of post-weaned piglets. Our study was committed to discover the positive influence of *L. salivarius* with a tailored concentration that may bring to the gut health and try to explore its role in the modulation of inflammation as well as oxidative stress after bacterial infection.

## Materials and Methods

### Bacterial Strains

The *L. salivarius* strains used in this study were isolated from the feces of healthy piglets. After purifying, *L. salivarius* was cultured in De Man, Rogosa and Sharpe (MRS) agar medium at 37°C for 24 h with an anaerobic environment. The culture solution was later centrifuged at 3,000 rpm for 10 min at 4 °C. The vacuum freeze-drying machine (Tofflon, Shang Hai, China) was used to acquire the bacterial powder in which 1 × 10^10^ CFU/g *L. salivarius* could be detected. The bacterial concentration was finally measured by an ultraviolet spectrophotometer (Nano Drop, Thermo Fisher, America) with the optical density being set at 550 nm.

### Animals, Diets, and Experimental Design

All pigs used in this experiment were born naturally at 114 days gestation which was considered as the full term pregnancy. A total of 72 crossbred healthy female piglets (Landrace × Yorkshiere × Duroc) were reared by sows and weaned at 21 ± 2 days of age. Piglets with initial body weight (BW) 6.44 ± 0.25 kg were randomly assigned to 1 of 4 treatments, each treatment with six replicates. All the subjects were housed in 6 pigpens with an ambient temperature of 20–30°C, and each pen was equipped with a feeder and a drinking nipple to allow piglets free access to food as well as drinking water. The corn-soybean meal-fish meal basal diet (Table 1) for the subjects was formulated to meet the estimating nutrient requirements released by the National Research Council (NRC 2012), and all the added ingredients stayed the same as those used in our previous study (28). Piglets were carefully weighed throughout the test by using the electronic weighing system. Through the entire growing period, the average daily gain (ADG) of each piglet was defined as the difference between the ending and beginning weight divided by feeding days. Meanwhile, the equation for the average daily feed intake (ADFI) was the total of feed used per pen day averaged by three piglets together with trial days.

**Table 1:** Composition and nutrient levels of experimental diets (%w/w, air-fed basis).

**Item**	**Amount (g/kg)**
Corn, yellow	63.20
Soybean meal, 43% CP (crude protein)	19.00
Whey powder	4.80
Fish meal, 65% CP	8.60
Glucose	1.00
Acidifier	0.30
Calcium hydrogen phosphate	0.60
Limestone	0.70
Salt	0.30
L-Lys∙HCL, 78% Lys	0.30
DL-Met, 99% Met	0.10
L-Thr, 98% Thr	0.10
Vitamin and mineral premix^a^	1.00
Calculated composition	
DE (digestible energy), Mcal/kg	3.25
Lys, %	1.39
Met, %	0.53
Analyzed composition	
Crude protein	18.72
Crude fat	3.38
Calcium	0.85
Total phosphorus	0.68
Crude fiber	2.15

a *Supplying a minimum per kilogram complete diet of: 12,500 IU Vitamin A; 1,250 IU Vitamin D; 125 IU Vitamin E; 90 μg Vitamin B12; 10 mg riboflavin; 48 mg pantothenic acid; 35 mg niacin; 4.5 mg folic acid; 0.25 mg biotin; 130 mg Fe; 180 mg Zn; 15 mg Cu; 30 mg Mn; 0.60 mg I and 0.25 mg Se*.

### Experimental Procedures

The selected piglets were randomly divided into four dietary groups: the basal diet (C), basal diet supplemented with 0.05% *L. salivarius* (T1), basal diet with 0.1% *L. salivarius* (T2), and basal diet with 0.2% *L. salivarius*. LPS from *E. coli* serotype O55:B5 (Sigma Chemical Co., St. Louis, MO) was intraperitoneally injected (200 μg/kg body weight) right after the 14-days trial. The applied dosage of LPS was referred to previous description in our lab (29) and the sterile 0.9% NaCl solution was used for diluting LPS to reach the confirmed concentration.

### Collections of Blood and Tissue Samples

After LPS injection, the blood samples (5 ml/piglet) of piglets (one piglet per pen) were collected from precava 6 h after intraperitoneal administration, from which the serum was later separated by centrifugation at 3,000 rpm for 20 min with the temperature of 4°C. The collected serum was transferred into microtubes and then stored at −80°C for the detection of cytokines and antioxidant levels. Sodium pentobarbital (50 mg/kg body weight) was delivered by a rapid intracardial injection for euthanizing the piglets and the jugular exsanguination procedures were also initiated to ensure the death. After sacrifice, certain tissue samples were obtained for the performances of quantitative PCR (qPCR) and western blotting. An ~10 cm segment of intestine was incised from the mid-jejunum, flushed with ice-cold saline and opened longitudinally. MLNs, spleens and livers were dissected out. All the above harvested samples were immediately immersed in liquid nitrogen and stored at −80°C for RNA isolation.

### Measurements of D-lactic acid, DAO, and CRF From the Serum

The enzyme activity of DAO and CRF levels were measured using enzyme-linked immunoassay kits (Jiancheng Bioengineering Institute, Nanjing, China). A chemical assay kit from the same company was purchased for D-lactic acid analysis. All the performances were based on the supplier's protocols.

### Determination of Cytokines From Serum by ELISA

The porcine enzyme-linked immunosorbent assay (ELISA) was employed for detecting a variety of soluble cytokines produced in the serum of piglets from designed four groups, including IL-1β, IL-6, IL-10, TNF-α, and TNF-γ (R&D Systems, Minneapolis, MN). All the reagents from the kit were placed in the room temperature for 15 min prior to performances. 50 μL of Assay Diluent was firstly added to each well and 50 μL of both high and low standard, control and tested serum were separately added per well. After mixing, the plate was covered with adhesive sealer at shaker for 2 h at room temperature. Each well was then aspirated and washed with 400 μL wash buffer four times. Next, 100 μL conjugates was gently added into each well and incubated for 2 h at the shaker with room temperature. Substrate solution, protected from light, was finally used for 30 min incubation, after which the stop solution was added. The result of optical density was collected through the micro-plate reader set to 450 nm.

### RNA Isolation

The frozen samples were replaced on ice to thoroughly thaw and the shredded parts were poured with 1,000 μL TRIzol for 5 min complete degradation of cell membrane and proteins. 200 μL chloroform was added to the cell lysate and the mixture was inverted several times. After inversion, the substances with three different layers were centrifuged at 12,000 g for 15 min at 4°C. The upper layer containing RNA was removed and mixed with 500 μL isopropanol and final pellet was obtained after centrifugation for 10 min with the previously mentioned rotational speed and temperature. 1 ml 75% ethanol served as the reagent for washing pellet which was finally air dried for 10–15 min and dissolved in RNase free water for the following analysis. Both the amount and the purity of RNA product were measured by Picodrop Microliter Spectrophotometer. In addition, the integrity of the total RNA was determined with 1% agarose gel electrophoresis.

### cDNA Synthesis and Quantitative PCR

cDNA was synthesized from the tissue-derived RNA with an amount of 2 μg. This reverse transcription included the use of MMLV-reverse transcriptase, 5X buffer, dNTP mix and RNasin ribonuclease inhibitor to a total volume of 25 μL under the guidance of instruction's protocol (Promega, Madison, WI). The qPCR was then conducted in triplicate on ABI 7500 Real-Time PCR detector with a SYBR Premix Ex Taq (Tli RNaseH Plus) and qPCR kit (TaKaRa Biotechnology, Inc., Shiga, Japan). The primer pairs for detected genes were referred to previous reports (30, 31) and the sequences are listed in Supplementary Table 1. The relative gene expression data were analyzed by normalizing the threshold cycle (Ct) value of each sample to that of GAPDH, which was calculated with 2^−ΔΔ*Ct*^ method.

### Biochemical Assay of MDA

The reagent mixture provided by commercial MDA Assay Kit (Nanjing Jiancheng Bioengineering Institute, Nanjing, China) and 200 μL serum were homogenized according to the manufacture's guidelines. Briefly, the mixture was boiled for 40 min in a water bath. After incubation and cooling, the solution was centrifuged at 4,000 rpm for 10 min. The obtained supernatant was pipetted for ultra-violet spectrophotometry. The intensity of the formed color, corresponding to the level of MDA, was read at 532 nm and the final concentration was determined by plotting the obtained value against standard curve.

### Estimation of Antioxidant Enzyme Activities From the Serum

The activity of SOD, CAT and GSH-Px were assessed by using SOD Assay Kit, CAT Assay Kit and GSH-Px Assay Kit (Nanjing Jiancheng Bioengineering Institute, Nanjing, China), respectively. All the procedures of each particular detection as well as the calculation were based on the manufacturer's instructions. Some main steps were described below. For determining SOD activity, 100 μL prepared-serum with kit provided reagent mixture were firstly vortexed and incubated in a 37°C water bath for 40 min. The presence of SOD could reduce the component of nitrite in the reaction and 2 ml color developing agent was later utilized to generate a particular reddish-purple compound whose absorbance was later determined spectrophotometrically at 550 nm. In order to evaluate CAT activity, 100 μL prepared-serum with mixed reagents were kept in a 37°C water bath for 60 s, after which the color developing reagent was added and a light-yellow product finally appeared after reaction. The maximum absorbance of this compound was colorimetrically measured at 405 nm. One unit of CAT activity was defined as the amount of enzyme required for destroying 1 μmole H_2_O_2_ per minute. GSH-Px activity was measured by orderly adding provided reagents into the prepared supernatant and 5 min was required for this reaction. The absorbance of the yellowish mixture was finally determined at 420 nm.

### Protein Extraction and Western Blotting

For protein extraction, about 50–100 mg intestinal tissue was firstly immersed in 1 ml NP-40 lysis buffer together with a cocktail of protease and phosphatase inhibitors. Obtained homogenates were then vortexed, and after which the lysates were subjected to a 12,000 × g rotational speed produced by centrifugation for 15 min at 4°C. After spinning, the supernatants containing proteins were collected for electrophoresis. Protein concentration was estimated by the Bradford protein assay, a simple spectroscopic technique, to make sure the amount of protein sample loaded in each well was kept constant. After electrophoresis, separated proteins on the gel were transferred onto polyvinylidene difluoride (PVDF) Membrane (Millipore). This membrane was then coated with blocking buffer and followed by incubation of primary antibodies for 2 h at room temperature. After several rinsing steps, the horseradish peroxidase (HRP)-conjugated secondary antibodies were applied on the same membrane for 1 h at room temperature. The Western blot luminescence detection kit (Santa Cruz Biotechnology, Santa Cruz, CA) was utilized to present protein bands after appropriate exposure by AlphaImager 2200 and the band densities were finally quantified. Antibodies used in this study were purchased from Abcam, including anti-Zo-1, anti-Occludin, anti-Claudin-1, and anti-β-actin.

### Statistical Analysis

All experiments were performed with six independent replicates. Statistical analysis was created by using SPSS v. 22.0, and differences in data were evaluated by one-way ANOVA with *p* < 0.05 being considered statistically significant.

## Results

### *L. salivarius* Exerted Growth-Promoting Effects in Piglets

Piglet fed diets containing 0.1% *L. salivarius* contributed to an improved growth 14 days after weaning as observed by the significant increase of ADG along with BW. Aside from the fairly consistent data of ADFI derived from four groups, the feed and gain radio (F/G) also showed no statistical significance but a tendency of reduction in all groups treated with *L. salivarius*. It's also worth noting that the diarrhea incidence was counted in each group and the acutely lower possibilities were attributed to 0.1 or 0.2% *L. salivarius* (Table 2).

**Table 2:** Potential effect of *L. salivarius* on growth performance and diarrhea incidence of weaned-piglets after LPS stimulation.

**Item**	**C**	**T1**	**T2**	**T3**	**SEM**	***P*-value**
BW (kg), d 0	6.43	6.44	6.44	6.45	0.18	1.000
BW (kg), d 14	16.58^b^	17.57^b^	18.8^a^	18.5^b^	0.39	0.179
ADFI (g)	521	548	567	562	28	0.945
ADG (g)	242^b^	265^b^	294^a^	287^a^	8	0.098
F/G	2.13	2.04	1.9	1.93	0.05	0.450
Diarrhea incidence, %	10.32^a^	7.94^ab^	5.16^b^	5.95^b^	0.78	0.080

a,b *Within a row, values without a common superscript letter differ significantly (P < 0.05)*.

### The Serum Level of D-Lactic Acid, DAO, and CRF After *L. salivarius* Ingestion

**Figure 1:**
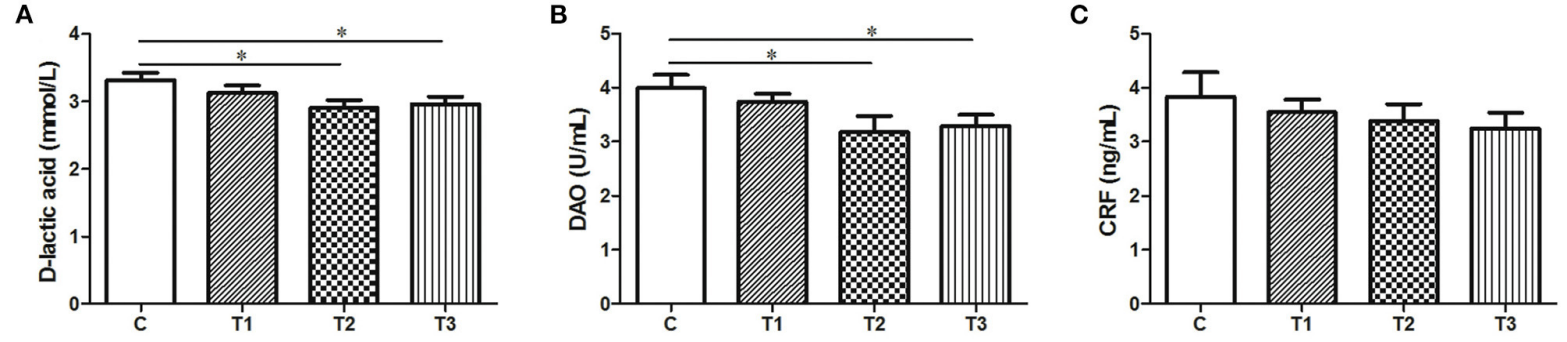
The effect of *L. salivarius* on intestinal permeability and CRF level in LPS-injected subjects during weaning transition. **(A)** D-lactic acid, **(B)** DAO, and **(C)** CRF were evaluated in the serum. *P < 0.05. Error bars plotted as mean ± SEM.

Both the D-lactic and DAO levels were obviously attenuated in response to either 0.1% or 0.2% *L. salivarius* (*P* < 0.05) (Figures 1A,B). However, no difference of CRF was presented in *L. salivarius*-supplemented groups when compared to the control one (Figure 1C).

### The Protective Effects of *L. salivarius* on Epithelial Integrity

**Figure 2:**
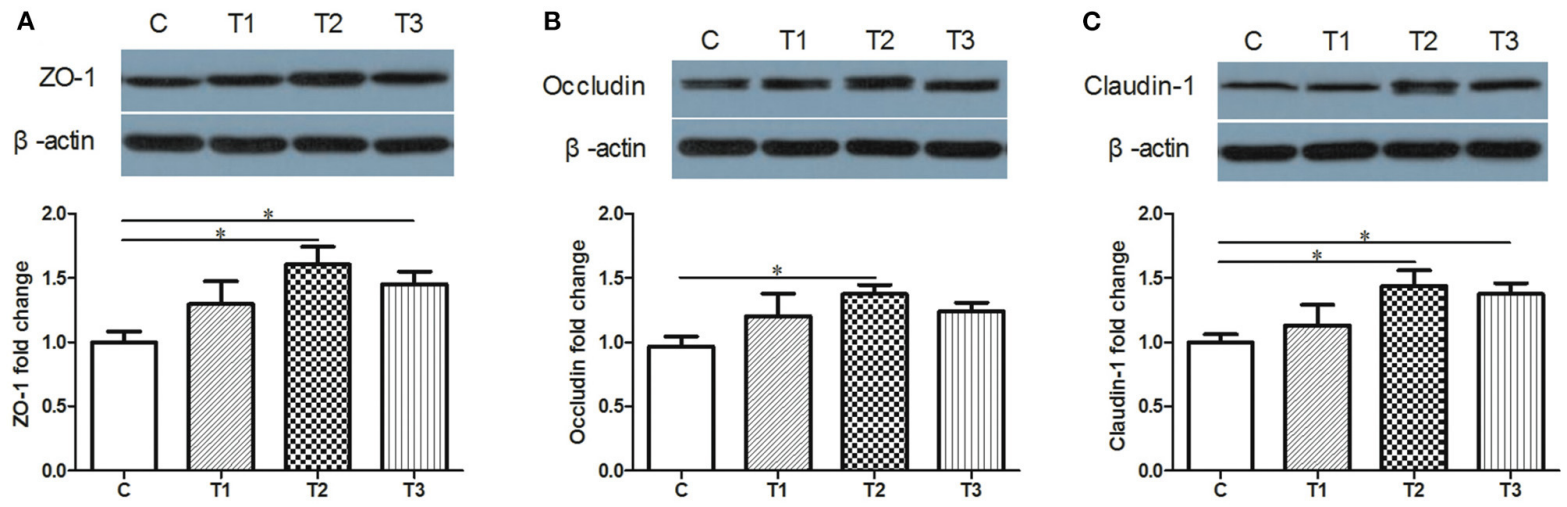
The positive influence of *L. salivarius* on the expression levels of tight junction proteins in post-weaned piglets after stimulated with LPS. **(A)** ZO-1, **(B)** Occludin, **(C)** Claudin-1 were detected in part of mid-jejunum by western blotting. β-actin, the housekeeping protein, was used as a loading control for proper interpretation. *P < 0.05. Error bars plotted as mean ± SEM.

Candidate proteins of tight junctions were analyzed by western blotting. Compared with the control group, an increased trend of ZO-1, Occludin and Claudin-1 were detected in *L. salivarius*-treated subjects regardless of the using amount (Figure 2). Specifically, ZO-1 and Claudin-1 were significantly increased by both 0.1 and 0.2% *L. salivarius* pretreatment (Figures 2A,C) while 0.1% *L. salivarius* exerted an obvious enhancement of Claudin-1 (*p* < 0.05) (Figure 2B).

### The Influence of *L. salivarius* on the Alternation of Pro-Inflammatory and Anti-Inflammatory Cytokines in the Serum

**Figure 3:**
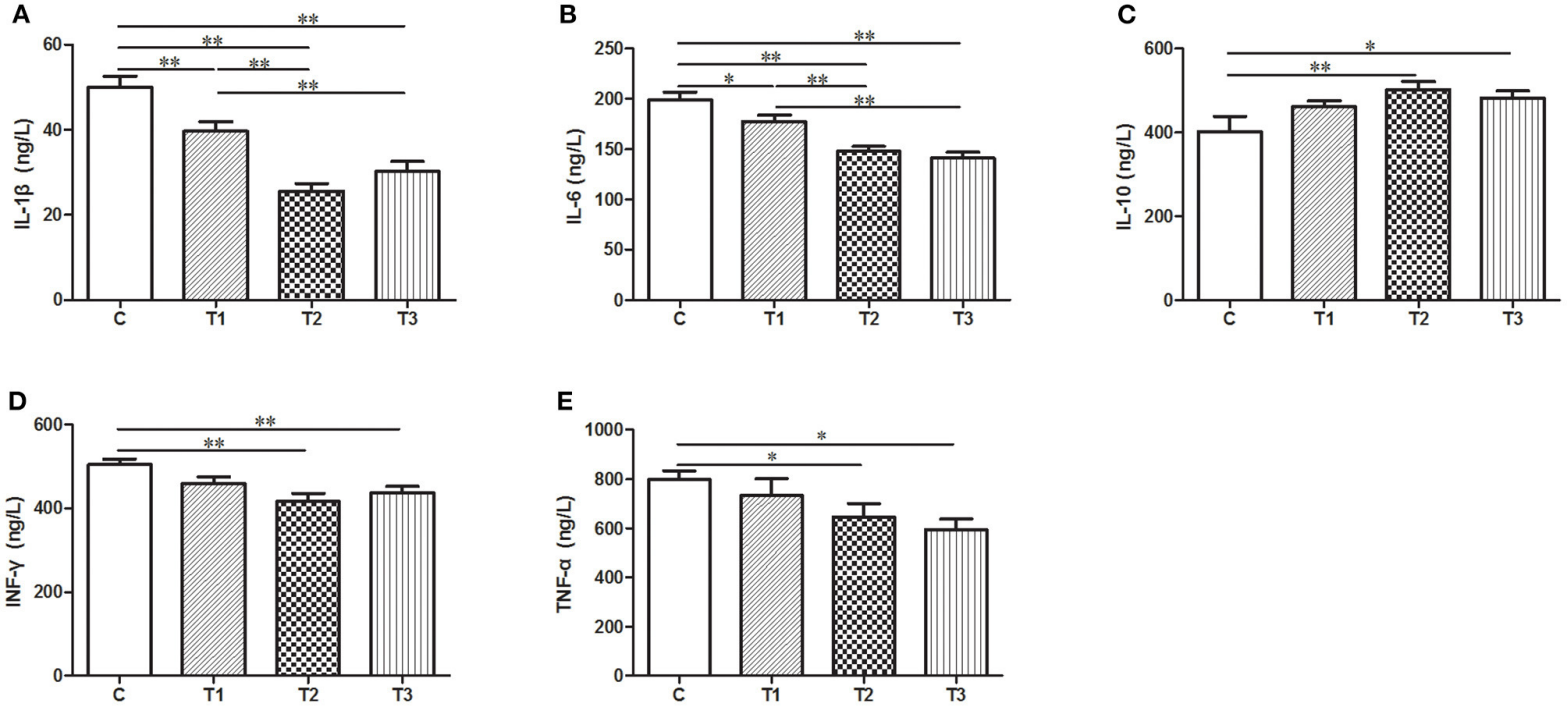
A wide range of pro- and anti-inflammatory cytokines were regulated following *L. salivarius* treatment on LPS-challenged individuals. The cytokine levels of **(A)** IL-1β, **(B)** IL-6, **(C)** IL-10, **(D)** IFN-γ, and **(E)** TNF were evaluated from the serum by ELISA. *P < 0.05; **P < 0.01. Error bars plotted as mean ± SEM.

In our effort to figure out the potential function of *L. salivarius* in inflammatory process, a group of cytokines secreted in the serum were determined by ELISA. After infection, the uptake of 0.1 or 0.2% *L. salivarius* enabled the levels of IL-1β, IL-6, IFN-γ, and TNF-α to be markedly down-regulated while the decreasing level of IFN-γ and TNF-α caused by 0.05% *L. salivarius* did not reach the statistical significance (Figures 3A,B,D,E). Meanwhile, a significant elevation of IL-10 was reported in almost all *L. salivarius* pretreated piglets. Still, we presented an exception that even though there was a marginally increasing trend of IL-10 triggered by 0.05% *L. salivarius*, the change was not obvious (Figure 3D). Generally speaking, the variation of each inflammation-related cytokine detected in our study was significantly altered by 0.1 or 0.2% *L. salivarius* instead of 0.05% dosage.

### The Abundance of TLR2 and TLR4 in Spleen and MLNs With *L. salivarius* Involvement

**Figure 4:**
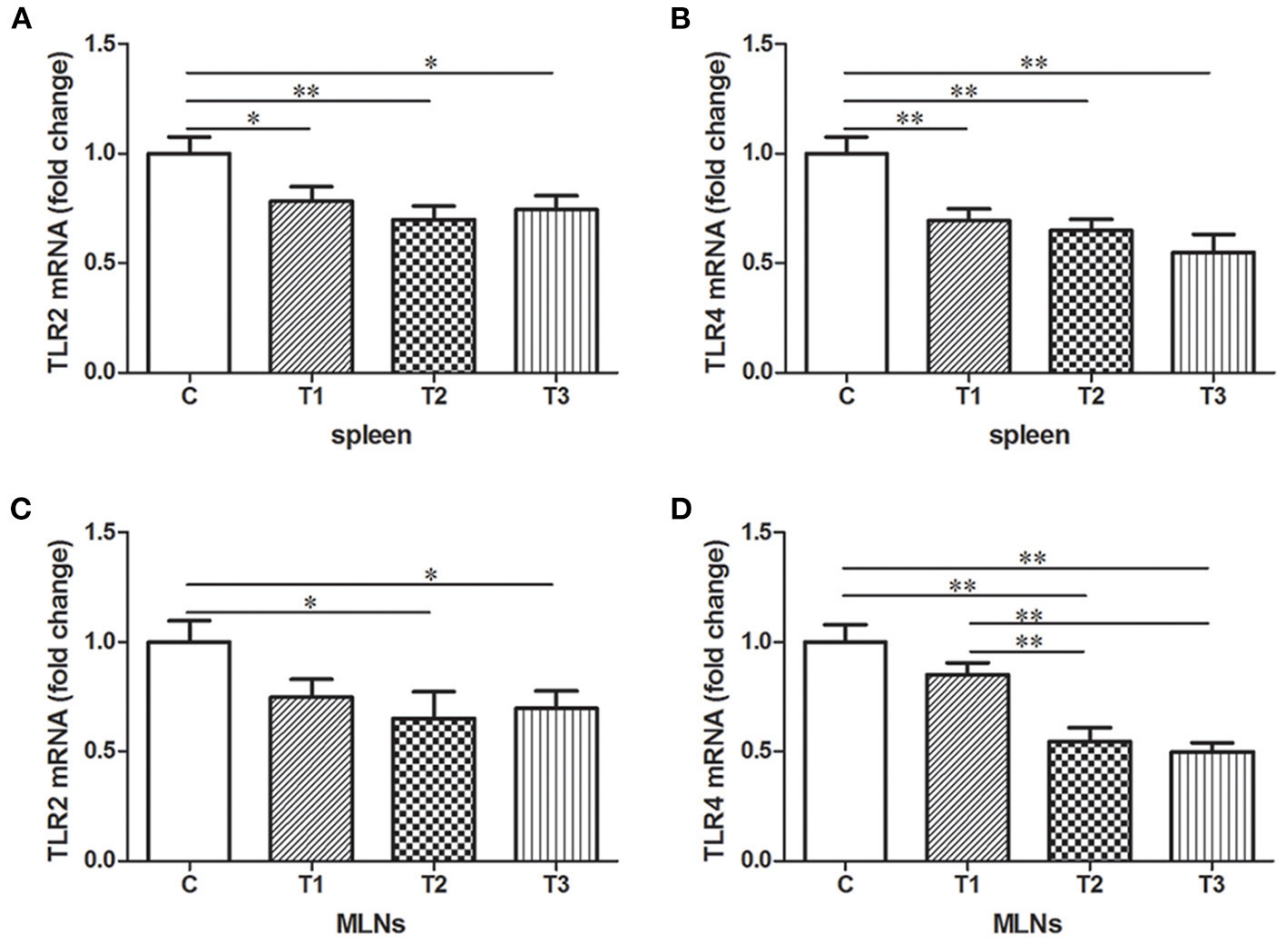
The down-regulated mRNA expression patterns of TLR2 and TLR4 induced by *L. salivarius* in spleen and MLNs of LPS-challenged piglets 14 days after weaning. The mRNA levels of **(A)** TLR2 and **(B)** TLR4 in spleen accompanied by **(C)** TLR2 and **(D)** TLR4 in MLNs were detected by qPCR. The relative gene expression data were analyzed by normalizing the threshold cycle value of each sample to that of GAPDH. *P < 0.05; **P < 0.01. Error bars plotted as mean ± SEM.

Expression of TLR2 and TLR 4 mRNA were examined in either spleen or MLNs isolated from the control and *L. salivarius*-treated piglets. In case of spleen, a decrease of TLR2 or TLR4 was found in groups treated with 0.05, 0.1, and 0.2% *L. salivarius*, respectively (Figures 4A,B). On the other hand, in MLNs, the level of TLR2 and TLR4 showed noticeable decrease only with the involvement of 0.1 or 0.2% *L. salivarius*. In fact, the alteration level of TLR2 and TLR4 in groups with 0.05% *L. salivarius* compared to that of the control was not quantitatively sufficient despite the tendency to decline (Figures 4C,D).

### The Modulation of Parameters Reflecting Oxidative Stress With *L. salivarius* Administration

**Figure 5:**
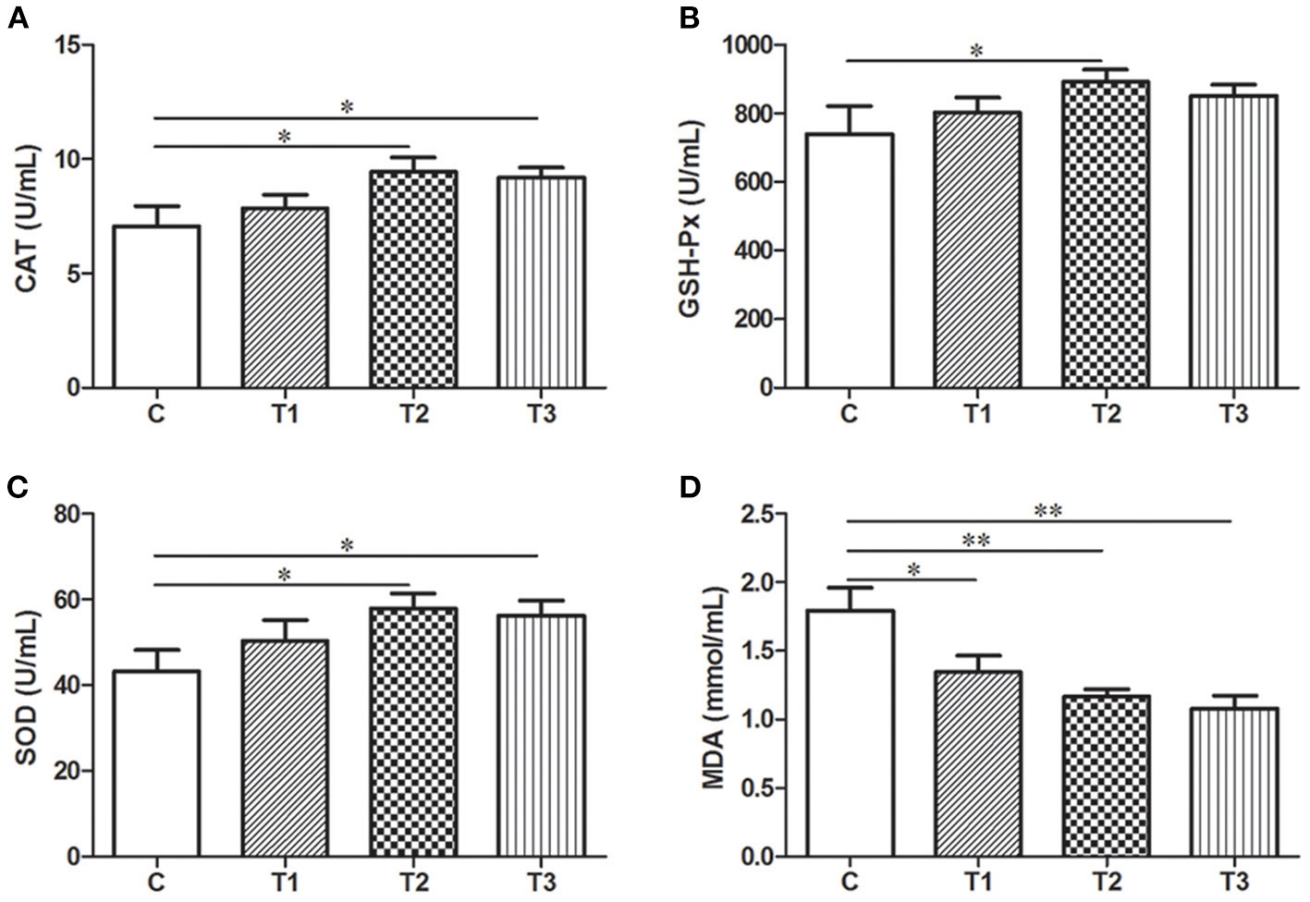
Biochemical alternations of indicators regarding oxidative stress in post-weaned piglets with LPS stimulation. **(A)** CAT, **(B)** GSH-Px, **(C)** SOD, and **(D)** MDA were detected in the serum. *P < 0.05; **P < 0.01. Error bars plotted as mean ± SEM.

As biochemical markers for oxidative stress in the body, the level of SOD, CAT, GSH-Px and MDA in the serum were examined by ELISA. *L. salivarius* administration with gradient concentration could all induce an elevation of oxidative enzymes as well as a markedly reduction of MDA compared to the control group (Figure 5). It's also worth mentioning that 0.1% *L. salivarius* had the best performance not only in significantly increasing SOD, CAT, and GSH-Px expression levels (*P* < 0.05), but also scavenging MDA (*P* < 0.01) (Figure 5).

### Quantitative Assessment of Potential Genes Related to Oxidative Stress in the Liver With Probiotic Treatment

**Figure 6:**
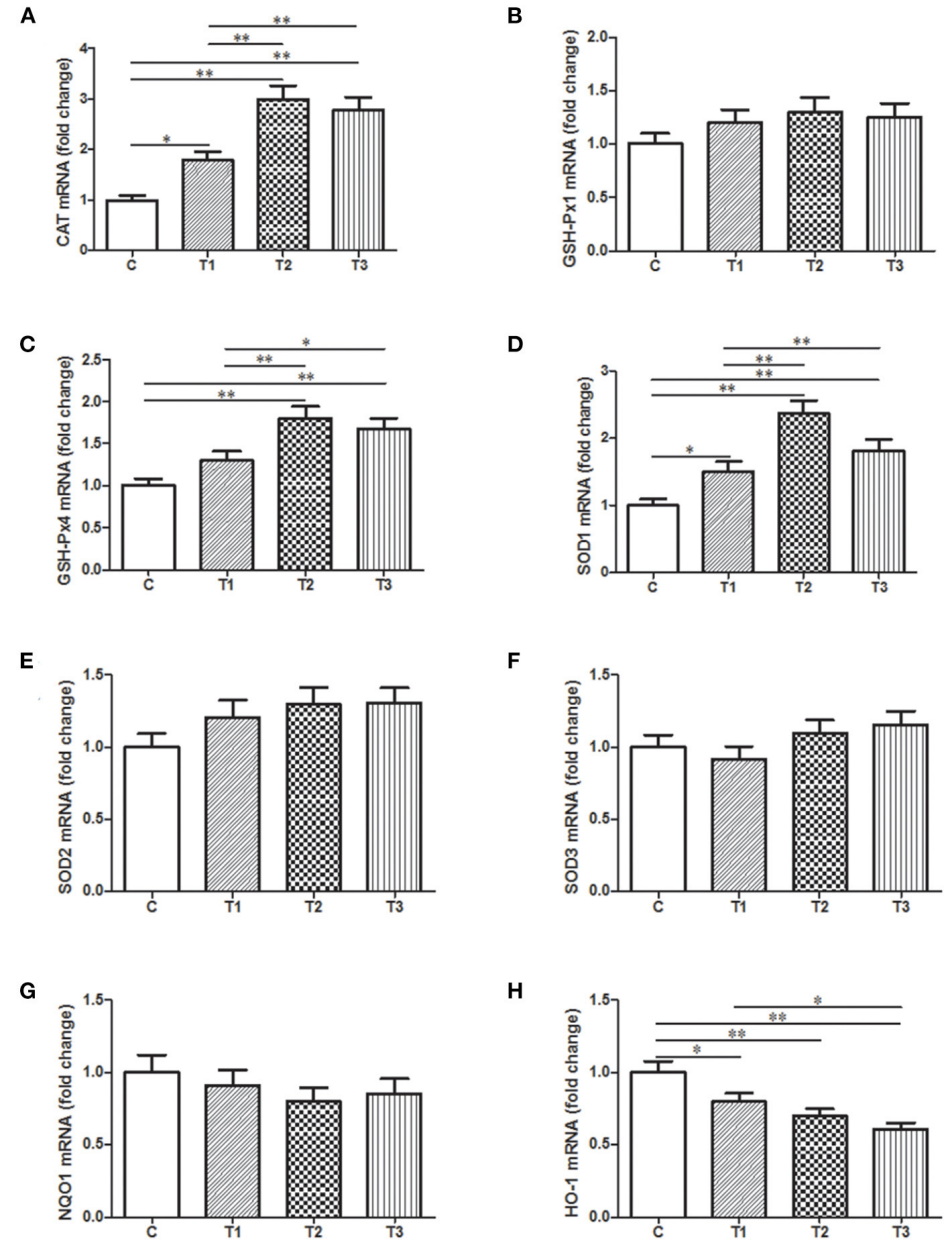
Relative mRNA expression levels of genes involved in the oxidation-related signaling pathways as well as the antioxidant system from the liver tissues of weaned-piglets. **(A)** CAT, **(B)** GSH-Px1, **(C)** GSH-Px4, **(D)** SOD1, **(E)** SOD2, **(F)** SOD3, **(G)** NQO1, and **(H)** HO-1 were measure by qPCR with GAPDH acting as reference gene for normalization. *P < 0.05; **P < 0.01. Error bars plotted as mean ± SEM.

In order to present a quantitative assessment of these oxidative stress markers, qPCR was performed with samples from the liver since it is a vital organ for detoxification and metabolism. There was a huge improvement of CAT mRNA expression in three *L. salivarius* involvement groups, like at least one-fold better than the control group (Figure 6A). Similarly, *L. salivarius* could also give rise to an enriched transcription of *sod1* and *gsh-px4* and the increasing level was significant. However, the incorporation of *L. salivarius* did not result in any noticeable change of SOD2, SOD3, and GSH-Px1 mRNA (Figures 6B,E,F). The mRNA level of HO-1 was significantly down-regulated in groups with *L. salivarius* treatment while no difference was noticed regarding to the expression level of NQO1 (Figures 6G,H).

## Discussion

The growth of piglets could be compromised due to a variety of posted challenges during the weaning period, such as a changing diet, a different surrounding setup, social interaction with other littermates and a higher incidence of getting viral or bacterial infection from pens. Researches posits that PWD is tied to the above-mentioned environmental or psychological factors, among which the ingestion of *E. coli* is always considered as the causal agent for intestinal disturbances with diarrhea being a common and severe clinical sign. Aside from an improvement of hygienic rearing condition, the diarrhea developed in post-weaned piglets is practically prevented by food additives, antibiotics, and oral vaccines (32). However, there is a report stating that due to the gut microbiota dysbiosis during early life, the vaccine efficacy could not be guaranteed based on the reduction of antibody levels (33). Since the excessive use of antibiotics have kept ongoing concerns about the development of antibiotic-resistant bacteria strains as well as the detrimental impact on human beings, the usage of antibiotics has been gradually minimized and even eliminated in EU since 2006 (34). Previously published scientific consensus in relation to distinct probiotics demonstrated that this category of live microorganisms could contribute to the enhancement of piglet enteric health after weaning (35). As a major component of probiotics, certain Lactobacillus strains have been delineated to function in gut health promotion. For example, the treatment of *Lactobacillus plantarum* is proposed as a feasible approach for improving diarrhea resistance and protecting intestinal morphology when young piglets are challenged by *E. coli* (36). In a similar study, the dietary addition of *Lactobacillus rhamnosus* GG (LGG) is also proved to help intestinal recovery of *E. coli*-challenged piglets (37). However, there is a noteworthy research stating that some risks associated with high-dose *L. rhamnosus* in an *E. coli* model of piglet diarrhea should not be underestimated (38). Since the exact mechanisms by which Lactobacillus confer benefits to the gut health are still limited, we here used *L. salivarius*, a newly separated strain belonging to the family of Lactobacillus, to target the infected individuals for exploring its efficacy in maintaining intestinal physiology and it remains to be seen whether the predicted efficacy based on a dose-dependent manner or not.

An LPS model was selected in our study to mimic the *E. coli* infection to which newborn piglets may be exposed in their normal living environment. Earlier results have proved that *Lactobacillus reuteri* D8 and *Lactobacillus delbrueckii* (39, 40) are conducive to the increased BW as well as decreased diarrhea rate, two promising signs of healthy status of intestine in which nutrient absorption usually occurs. Here in our work, it was remarkable to mention that 0.1% *L. salivarius* brought the maximum BW and ADG as well as the minimum diarrhea incidence among three treated groups (Table 2), indicating an advantageous effect of this strain with suitable dosage on the growth performance of post-weaned piglets.

Tight junctions of intestinal mucosa serve as a multifunctional assemble residing between epithelial cells. In fact, this complicated structure contains a cluster of proteins and their quantity and distribution appear to have impacts on the integrity of intestinal barrier and functional permeability (41, 42). As noted previously, the increased permeability in colitis was preceded by Dextran sulfate sodium via the loss of ZO-1, facilitating inflammatory infiltrate (43). In this case, there is also higher possibility for certain substances in the lumen to leak into the surrounding blood due to the increased permeability, such as pathogens. To our knowledge, the intestinal wall of piglets tends to be incomplete during their early days (44), so investigators have been trying to figure out possible solutions to improve this discontinuity. For instance, the up-regulated levels of Occludin and ZO-1 induced by either *Lactobacillus reuteri* I5007 or *L. plantarum* are conducive to the improved function of the intestinal mucosal barrier (36, 45). Therefore, it appears justifiable that *L. salivarius* has a potential role in making compensation for the impaired intestine because in this study, the abundance of tight junction Occludin, claudin-1 and ZO-1 were significantly augmented with actions of this probiotic (Figure 2). Since D-lactic acid and DAO normally present in lumen and villous cells, respectively (46), these two substances were able to indirectly reflect intestinal status after damage. Therefore, the decreased levels of D-lactic acid and DAO in groups with *L. salivarius* involvement (Figure 1), as demonstrated in our study, could be seen as powerful evidence for showing the ameliorated barrier after damage. Another interesting finding of this part is that low additive of *L. salivarius* barely had any difference until 0.1% *L. salivarius* started to markedly help synthesize tight junction proteins as well as drop the level of D-lactic acid and DAO, which is in line with the fact that this particular dosage was also able to maximize the growth performance of piglets (Table 2). This specific probiotic strain seems to confer protection against intestinal damage caused by bacterial infection and a particular dosage, 0.1%, may display the most effective potency in optimizing the tight junctions when common infection occurs.

In fact, a para-cellular seal might not be amply acquired with the existence of Occludin and ZO-1 (47), which means other proteins, like junctional adhesion molecules (8), still await characterization. Our study also presented a negligibly decreasing trend of CRF from the serum (Figure 1C). One previous review has hypothesized the disturbance of the brain-gut axis could lead to dysfunction of intestine in both human and animal models via emitting CRF systemically or peripherally (48). Alongside the harmful pathogens, it has been acceptable that side effects on gut could be produced due to the psychological stress from which piglets are more likely to suffer after weaning. The relationship of stress and the regulation of gastrointestinal permeability has been thoroughly summarized in the same review as well (48), so it was no wonder that a slightly decline tendency of CRF was observed in probiotics-added groups.

The immune system is responsible for distinguishing invading microorganisms depending on a sophisticated recognizing system. In general, TLRs and NOD-like receptors (NLRs) feature in identifying particular components of pathogens and then activate a bunch of signaling pathway for inflammatory response. Once this activation initiates, a cascade of immunostimulatory cytokines, such as IL-1β, IL-6, IL-10, IFN-γ, and TNF, will be initiated to amplify the inflammatory reaction whereas the opposite consequence brought by immunosuppressive cytokines like IL-10 is to limit the inflammation. From this perspective, the protective effects of probiotics on the inflammatory process in piglets have been illustrated by some investigators. *Lactobacillus acidophilus* and LGG could counteract gut inflammatory response to ETEC by down-regulating pro-inflammatory cytokines while increasing anti-inflammatory regulators via MAPK/NF-κB signaling pathway (28, 49). Selected Lactobacilli is suggested to modulate TLR4 signaling in intestinal cell lines, resulting in the suppression of inflammation (50). *L. plantarum* N14 has the immunoregulatory capacity to change the pro-inflammatory cytokines in porcine intestinal epithelial cells in response to ETEC challenge via TLR2 (51). Here in our study, it turned out that IL-1β, IL-6, IFN-γ, and TNF-α were significantly potentiated by 0.1 and 0.2% *L. salivarius*. In contrast, the expression levels of IL-10 shown in piglets fed with 0.1 and 0.2% *L. salivarius* was significantly higher (Figure 3). In agreement with current knowledge, the improved permeability of intestinal barrier might be achieved when the adding concentration of *L. salivarius* reaches 0.1% and this strain is beneficial for partially ameliorating the LPS-induced inflammatory response.

Spleen and MLNs, defined as the major immunological sites, have crucial capability of maintaining homeostasis in the organism. An enormous number of macrophages placed in these two locations have prominent roles in scavenging blood-borne pathogens and activating adaptive immunity with the support of TLRs. One research stated that the absence of spleen in the body may lead to an impaired host defense response after pneumococci infection (52). In case of spleen or MLNs, both TLR2 and TLR4 were observed significantly higher in control groups compared to groups treated with 0.1–0.2% *L. salivarius* (Figure 4), which indirectly indicated the contribution made by *L. salivarius* to ease the inflammation. One previous research posits that the presence of *L. acidophilus* significantly reduces the level of TLR2 and TLR4 in both spleen and MLNs, the data of which is consistent with ours (28). Meanwhile, we also assume that the decreased level of TLR2 and TLR4 in both spleen and MLNs could be considered as a result from *L. salivarius* improving intestinal barrier after infection since the pathogens leaked from lumen might be reduced. Macrophages from MLNs and spleen might be recruited to the infectious sites and start to engulf foreigners via identifying the membrane TLRs. Our data provide us with another clue that thanks to the *L. salivarius* with applicable dosage, the dampened pro-inflammatory cytokines in the serum might make the attraction of these macrophages unnecessary. Nevertheless, the definite mechanism by which *L. salivarius* might be involved in inhibition of this movement remains vague and additional exploration is still required to obtain a complete understanding.

It's obvious that the dynamic balance between ROS and antioxidants is kept normal under regular physiological condition. When functioning improperly, free radicals will interact with available biological molecules in the body and thus act as an inducer of oxidative stress. In this case, antioxidants are generated to function in detoxifying the reactive ROS and thus conferring oxidative stress resistance. The initiation of this defense will activate a classic redox-sensitive signaling pathway, in which erythroid 2-related factor 2 (Nrf2) will detach from kelch-like ECH-associated protein-1 (Keap1) in the cytoplasm and then translocate into the nucleus as a transcription factor. The regulation of Nrf2 on heme oxygenase-1 (HO-1) and NADPH quinineoxidoreductase-1 (NQO1) is responsible for an assortment of enzymatic or non-enzymatic compounds as antioxidants (53). For instance, SOD, CAT and GSH-Px are defined as antioxidant enzymes and have been taken for granted in numerous researches as the biomarkers for oxidative defense. Four types of GSH-Px have been discovered to be responsible for the reduction of superoxide radicals and lipid hydroperoxides (54), among which GSH-Px1 and GSH-Px4 are most widely investigated. The unbalanced ROS in the body could be the target for these antioxidant enzymes which consequently render the oxidative stress suppressed. However, these native enzymatic substances sometimes are not enough to protect the living creatures from oxidative damage. New interests concerning antioxidant capacity of probiotics in protecting creatures against oxidative damage have been constantly aroused. Multispecies probiotic supplementation is able to relieve oxidative stress in individuals with typeII diabetes, presenting a potent pharmacotherapeutic action (55). *Bacillus* SC06 could relieve the oxidative stress-related intestinal disorders in rats by reversing diquat-induced MDA augmentation as well as increasing GSH-Px activity (56). Up-regulation of antioxidant enzymes (SOD, CAT, and glutathione S-transferase) resulted from *L. plantarum* AS1 in the colon of cancer-bearing subjects suggests an anticancer benefit of this probiotic through its antioxidant attributes (57). In case of piglets, since previous studies have shown that weaning or diet contamination could cause the disordered intestine of piglets where the occurrence and the development of oxidative stress are common (58, 59). Dietary supplement including some chemical complexes or microorganisms have been reported to have positive influence on the oxidative stress (59). For instance, Baicalin-zinc or -copper is capable of exerting antioxidant capability in deoxynivalenol-challenged piglets, which is reflected in the reduction of HO-1, NQO-1, NF-κB (60, 61). One week after weaning, there is a decrease of SOD and GSH-Px activities as well as an increase of MDA, reflecting an imbalanced redox status during this stage (58). Under this circumstance, an improved antioxidant capability of weaned piglets is observed after the addition of *L. plantarum* by promoting of SOD, CAT, and GSH-Px activities (62). From a practical perspective, we aim to explore the mode of action of *L. salivarius* in ameliorating oxidative issues occurring in weaned piglets. Here in our study, *L. salivarius* brought a significant increase in serum levels of SOD, CAT, and GSH-Px coupled with a marked decrease of MDA in post-weaned piglets (Figure 5), the final product of lipid peroxidation and a commonly used lipid marker of oxidative stress as well. To further validate, quantitative assessment was evaluated on genes encoding these cytoprotective proteins by qPCR (Figures 6A–F) and the analysis showed that the mRNA level of SOD1, GSH-Px4, CAT, and HO-1were highly augmented by the pretreatment of 0.1–0.2% *L. salivarius* (Figures 6A,C,D,H). Presumably, a protective effect of *L. salivarius* could be exerted against oxidative stress in LPS-treated piglets by the promoted expression of SOD1, GSH-Px4, and CAT via Nrf2/HO-1 pathway.

The reciprocal interaction between inflammation and oxidative stress is not something new, which has been witnessed for past decades by many investigators. There has been reached a consensus that oxidative damage is a major contributor to the decreased immune function, as thoroughly demonstrated in a review (63). Nevertheless, there has been a gap in our knowledge as to the mutual crosstalk between *L. salivarius*, inflammation and oxidative. We wonder there might be a possibility for *L. salivarius* to depress the inflammation firstly through assisting in the relief of oxidative stress, but this assumption required further theoretical and practical evidence.

## Conclusion

As alternatives to antibiotics and other species of probiotics, the supply of relatively high dose *L. salivarius* (0.1–0.2%) could bring positive contribution to the intestinal permeability through increasing tight junction proteins as well as inhibiting the leak of D-lactic acid and DAO in piglets challenged with LPS. Meanwhile, it appears justifiable that the pretreatment of *L. salivarius* possesses a feasible role in depressing the immune response by balancing the expression level of pro- or anti-inflammatory cytokines. We also speculate this weaken immunity might partially result from the effective instrument of *L. salivarius* on oxidant defensive process, during which the expression levels of SOD1, GSH-Px4, and CAT might be positively regulated via Nrf2/HO-1 pathway. In summary, the integrity of intestinal barrier in post-weaned piglets was restored through the suppressed immune response and oxidative stress after infection. This research provides new novel sights into the mechanism of probiotics involved in the intestinal improvement and the thorough understanding is the goal for treating or preventing intestinal diseases developed in post-weaned piglets.

## Data Availability Statement

The original contributions presented in the study are included in the article/Supplementary Materials, further inquiries can be directed to the corresponding author/s.

## Ethics Statement

The animal study was reviewed and approved by Animal Care and Use Committee of College of Life Science in Tianjin Normal University.

## Author Contributions

JQ and HL conceived and designed the study. ZS and YL performed the experiment. JQ analyzed the data and completed the manuscript. All the authors have read and approved the final manuscript.

## Conflict of Interest

The authors declare that the research was conducted in the absence of any commercial or financial relationships that could be construed as a potential conflict of interest.
