# Supplementary material for: Lactobacillus salivarius, a Potential Probiotic to Improve the Health of LPS-Challenged Piglet Intestine by Alleviating Inflammation as Well as Oxidative Stress in a Dose-Dependent Manner During Weaning Transition
Source: Front Vet Sci. 2020 Dec 16;7:547425. doi: 10.3389/fvets.2020.547425 (PMC7772421; doi:10.3389/fvets.2020.547425)
Supplement: Supplementary file 1 [file Table_1.DOCX]

Supplementary Material

# Supplementary Data

## Supplementary Table S1

**Table S1** Sequences of paired primers for target genes in qPCR

| **Genes** | **Sequence (5’ to 3’)** |
| --- | --- |
| *cat* | F: ACGCCTGTGTGAGAACATTG |
|  | R: GTCCAGAAGAGCCTGAATGC |
| *gsh-px1* | F: AAATGCTCACCCGCTCTTC |
|  | R: GTCATTGCGACACACTGGAG |
| *gsh-px 4* | F: CACCCTCTGTGGAAGTGGAT |
|  | R: TCACCACACAGCCGTTCTTA |
| *sod1* | F: TCCATGTCCATCAGTTTGGA |
|  | R: AGTCACATTGCCCAGGTCTC |
| *sod2* | F: TGGAGGCCACATCAATCATA |
|  | R: TTTCGAAGGAACCAAAGTCG |
| *sod3* | F: ACGCTGCTCTGTGCTTACCT |
|  | R: CTGCCAGATCTCCGTCACTT |
| *nqo1* | F: CCAGCAGCCCGGCCAATCTG |
|  | R: AGGTCCGACACGGCGACCTC |
| *Ho-1* | F: AGGCTGAGAATGCCGAGTTC |
|  | R: TGTGGTACAAGGACGCCATC |
| *gapdh* | F: GAAGGTCGGAGTGAACGGAT |
|  | R: CATGGGTAGAATCATACTGGACA |
